# Synthesis of Pillar[5]arene- and Phosphazene-Linked Porous Organic Polymers for Highly Efficient Adsorption of Uranium

**DOI:** 10.3390/molecules28031029

**Published:** 2023-01-19

**Authors:** Xiaoxiao Zhao, Ziyi Liu, Shuguang Zhang, Mehdi Hassan, Chunxin Ma, Zhenzhong Liu, Weitao Gong

**Affiliations:** 1 School of Chemical Engineering, Dalian University of Technology, Dalian 116024, China; 2 Department of Chemistry, University of Baltistan, Skardu 16100, Pakistan; 3 State Key Laboratory of Marine Resource Utilization in South China Sea, Hainan University, Haikou 570228, China; 4 Research Institute of Zhejiang University-Taizhou, Taizhou 318000, China

**Keywords:** uranium adsorption, seawater, porous organic polymers, pillar[5]arene, hexachlorophosphate

## Abstract

It is crucial to design efficient adsorbents for uranium from natural seawater with wide adaptability, effectiveness, and environmental safety. Porous organic polymers (POPs) provide superb tunable porosity and stability among developed porous materials. In this work, two new POPs, i.e., HCCP-P5-1 and HCCP-P5-2 were rationally designed and constructed by linked with macrocyclic pillar[5]arene as the monomer and hexachlorophosphate as the core via a macrocycle-to-framework strategy. Both pillar[5]arene-containing POPs exhibited high uranium adsorption capacity compared with previously reported macrocycle-free counterparts. The isothermal adsorption curves and kinetic studies showed that the adsorption of POPs on uranium was consistent with the Langmuir model and the pseudo-second-order kinetic model. Especially, HCCP-P5-1 has reached 537.81 mg/g, which is greater than most POPs that have been reported. Meanwhile, the comparison between both HCCP-P5-1 and HCCP-P5-2 can illustrate that the adsorption capacity and stability could be adjusted by the monomer ratio. This work provides a new idea for the design and construction of uranium adsorbents from macrocycle-derived POPs.

## 1. Introduction

With the traditional energy resources depleted, the universal need for nuclear energy is emerging rapidly as a sustainable energy source with no emission of greenhouse gases and unlimited energy proficiency [1,2]. Uranium is the main fuel resource for the nuclear industry and plays a dominating role in the field of energy production [3]. However, as the major contaminant of nuclear waste, uranium causes long-term harmful effects on human health and the ecological system [4]. In addition, the estimated quantity of uranium in seawater is 4.5 billion tons which is about one thousand times greater than terrestrial uranium [3]. Thus, currently designed and developed new adsorbents with proficient uranium extraction from aqueous solutions, i.e., seawater and nuclear industry wastewater have become a burning scientific issue in the field of energy production and environmental perspective [5,6].

Recently, various approaches were used for uranium extraction from aqueous solutions, i.e., membrane filtration [7], ion exchange [8], electrochemical [9], solvent extraction [6], and adsorption [10,11,12,13]. In these processes, adsorption shows great potential in uranium separation and recovery because of its environmental friendliness, easy operation, low cost, and wide adaptability. Furthermore, numerous varieties of adsorbing materials have also been developed and applied for the extraction of uranium, e.g., inorganic oxides [14,15], carbon materials [16,17] biomass materials [2,5,18], and metal–organic frameworks [19,20,21]. However, these materials exhibited several limitations such as relatively high expensive, less adsorption capacity, low selectivity, slow adsorption kinetics, and poor adsorbent stability.

In addition, among developed porous materials, porous organic polymers (POPs) receive widespread attention with high surface area, excellent porosity, and stability [22,23,24,25,26,27]. Therefore, it is necessary to design and develop new and efficient porous organic polymer materials for rapid, efficient, and stable adsorption of uranium. By tailoring the properties of diverse monomers and linkers, many fascinating POPs have been explored to exhibit impressive uranium adsorption capability [28,29,30,31,32]. As a new-generation supramolecular macrocyclic compound, pillar[n]arene has great advantages as a functional monomer to be incorporated into POPs to afford win–win merits, such as simple synthesis with the inherent electric-rich cavity, diverse post-synthetic strategies, and multi-stage pore structure i.e., fixed and cross-linked pores [33,34,35,36,37,38,39]. Despite several pillararene-based POPs that have been demonstrated with applications potential in the field of adsorption and separation [40,41,42,43,44], studies on their uranium adsorption properties are still unexplored.

Herein, to obtain stable and efficient pillararene-based POPs adsorbent materials, we chose highly stable hexachlorophosphonitrile (HCCP) as the linker and pillar[5]arene (P5) macrocycle as the monomer to fabricate two new POPs (HCCP-P5-1 and HCCP-P5-2) by a macrocycle-to-framework strategy. The P–O bond formed by the polymerization of HCCP and the hydroxyl groups of P5 is stable enough to form a highly tolerant framework [11]. Meanwhile, the nitrogen-rich structure in HCCP and the multiple hydroxyl groups bearing in the P5 structure can collaboratively bind to the 5f orbitals of the actinides, which can confer higher selectivity of the polymer [6]. The structural, physical, and chemical properties of both POPs have been studied in detail. The adsorption of both POPs for uranium in pure water and simulated seawater were studied systematically. As a result, both pillar[5]arene-containing POPs exhibit high uranium adsorption capacity compared with previously reported macrocycle-free counterparts. Especially, HCCP-P5-1 has reached 537.81 mg/g which is greater than most POPs that have been reported. The distinct difference between HCCP-P5-1 and HCCP-P5-2 is derived from the ratios between monomer P5 and linker HCCP, which gives rise to a tunable adsorption capacity and stability of both polymers. We believe this macrocycle-to-framework strategy could provide new insight into the design and construction of highly efficient uranium adsorbents.

## 2. Results and Discussion

### 2.1. Characterization

**Scheme 1:**
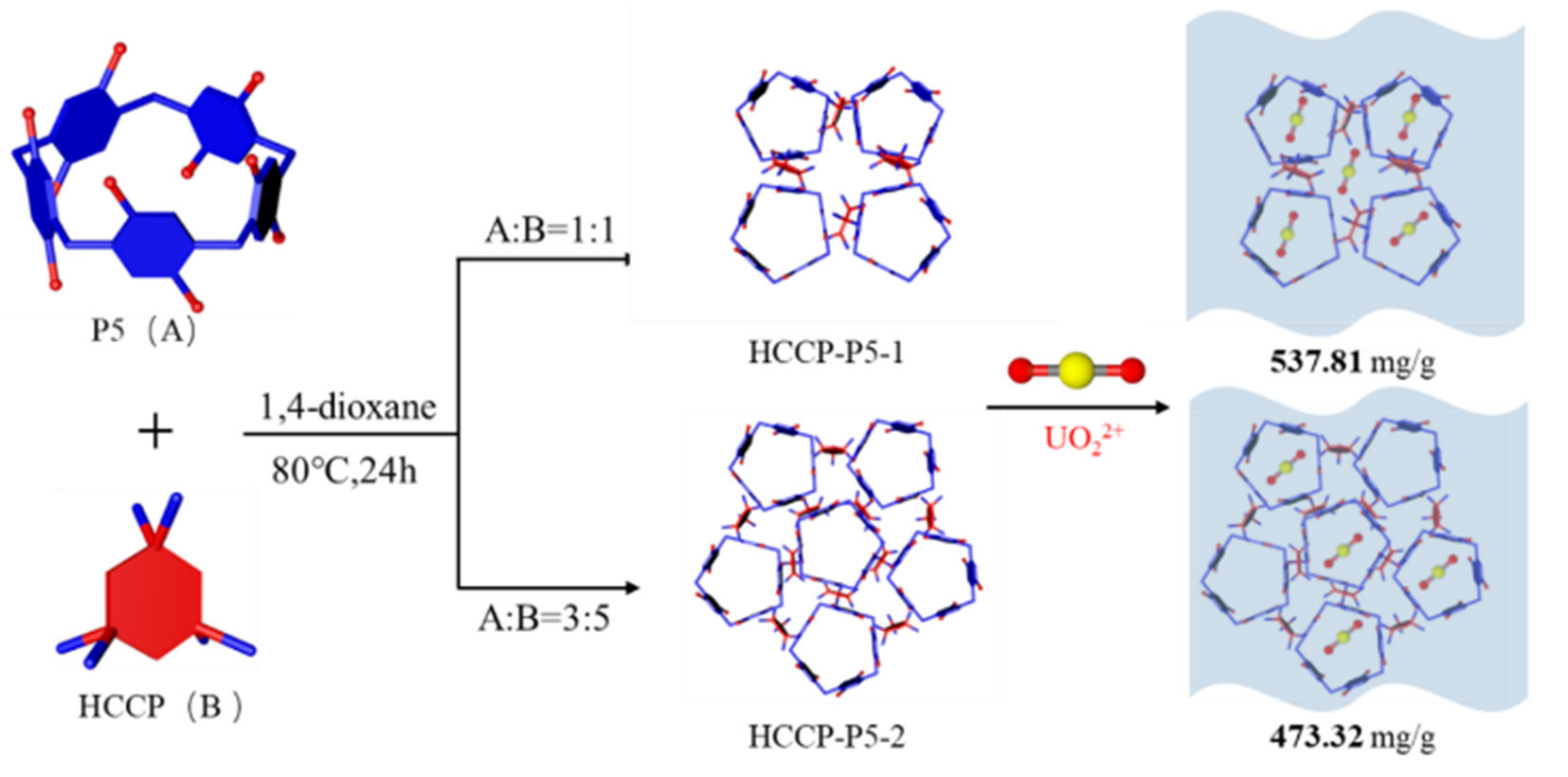
The illustration of pillar[5]arene-based HCCP-P5-1 and HCCP-P5-2 for efficient uranium adsorption.

**Figure 1:**
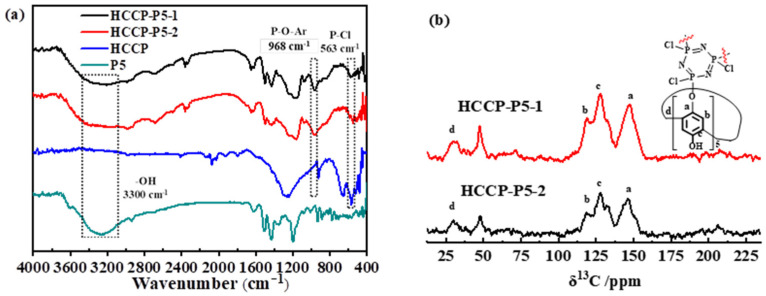
(**a**) FT-IR spectra of *P5*, HCCP-P5-1, and HCCP-P5-2; (**b**) ^13^C CPMAS NMR spectra and the structure of HCCP-P5-1 and HCCP-P5-2.

Both porous organic polymers, i.e., HCCP-P5-1 and HCCP-P5-2 were successfully synthesized by one-step polymerization (Scheme 1). FT-IR and ^13^C solid-state NMR confirmed the successful formation of the polymers. As presented in Figure 1a, the infrared spectra of the POPs and monomers displayed a broad peak at 3300 cm^−1^ corresponding to –OH, while a peak at 1234 cm^−1^ represents the P–O–Ar bond and a weak signal of P-Cl exhibited at 563 cm^−1^. Moreover, ^13^C solid-state NMR spectra (Figure 1b) showed significant signals at 28.08 ppm, 126.04 ppm, and 146.57 ppm, which implied the presence of pillar[5]arene macrocycle in both polymers.

Thermal stabilities of HCCP-P5-1 and HCCP-P5-2 were examined by thermal gravimetric analysis (TGA) presented in Figure S1. The TGA experiment exhibited four steps of weight loss. The first step recorded at less than 200 °C means the loss of adsorbed water and residual solvent. The second step of weight loss was noted down at lower 480 °C which is 21% for HCCP-P5-1 and 27% for HCCP-P5-2, respectively. This outcome can be ascribed to the elimination of the phosphazene moiety. In the third step weight loss was observed at 480 to 550 °C which indicated about 9% and 7% for both polymers which may be due to P–O bond cleavage. In the fourth weight loss step, the HCCP-P5-1 and HCCP-P5-2 were detected at over 550 °C which could be due to carbonization of the residual benzene ring of the core framework.

The morphology of polymers HCCP-P5-1 and HCCP-P5-2 were observed by using scanning electron microscopy (SEM), and both polymers showed irregular particles and loose porous states presented in Figure S2. Further, XRD analysis has no substantial characteristic of diffraction peaks which demonstrated that both POPs exhibited amorphous solid features (Figure S3).

**Figure 2:**
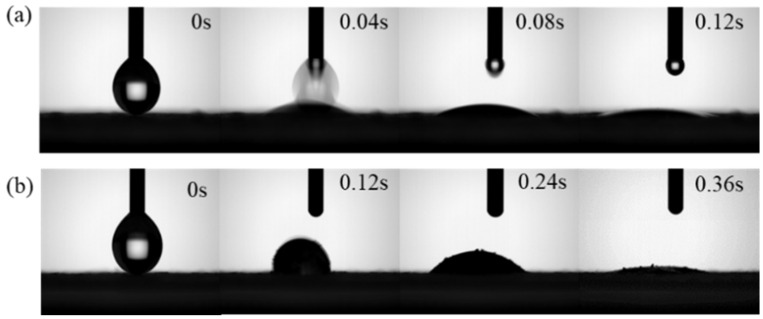
The surface contact angle of polymer (**a**) HCCP-P5-1, (**b**) HCCP-P5-2.

The hydrophilicity of HCCP-P5-1 and HCCP-P5-2 were recorded by the surface contact angle test. As shown in Figure 2, both polymers exhibited good hydrophilic capability which is rather critical for them as absorbent materials. Furthermore, the time for HCCP-P5-1 to reach the minimum contact angle is shorter compared with that of HCCP-P5-2, which indicated that HCCP-P5-1 has a higher hydrophilic property with more hydroxyl groups. This result implied that the hydrophilicity of both polymers can be easily tuned by adjusting the ratio between monomers P5 macrocycle and HCCP.

**Figure 3:**
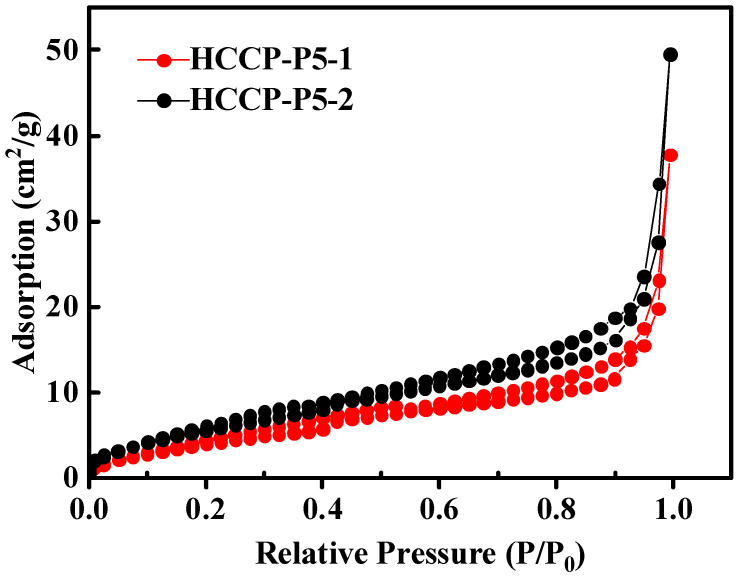
Nitrogen adsorption–desorption isotherm of HCCP-P5-1 and HCCP-P5-2 at 77 K.

The permanent porosity and specific surface area of HCCP-P5-1 and HCCP-P5-2 were further examined and calculated by using a nitrogen adsorption–desorption isotherm at 77 K. As shown in Figure 3 and Table 1, the specific surface area of HCCP-P5-1 and HCCP-P5-2 were recorded at 17.12 m^2^/g and 23.3 m^2^/g, respectively, while the main hole sizes of both polymers were observed as 4.728 nm. Both POPs are mesoporous materials, and although the BET is relatively low, the multi-hydroxyl structure of POPs makes the materials have a strong diffusion ability in the water, which accelerates the rapid contact between POPs and uranium in water. In addition, POPs are rich in nitrogen which makes POPs have a strong binding ability with uranium, so POPs have a strong adsorption ability to uranium although the surface area is small.

### 2.2. Adsorption Experiments

#### 2.2.1. Effect of Acidity

**Figure 4:**
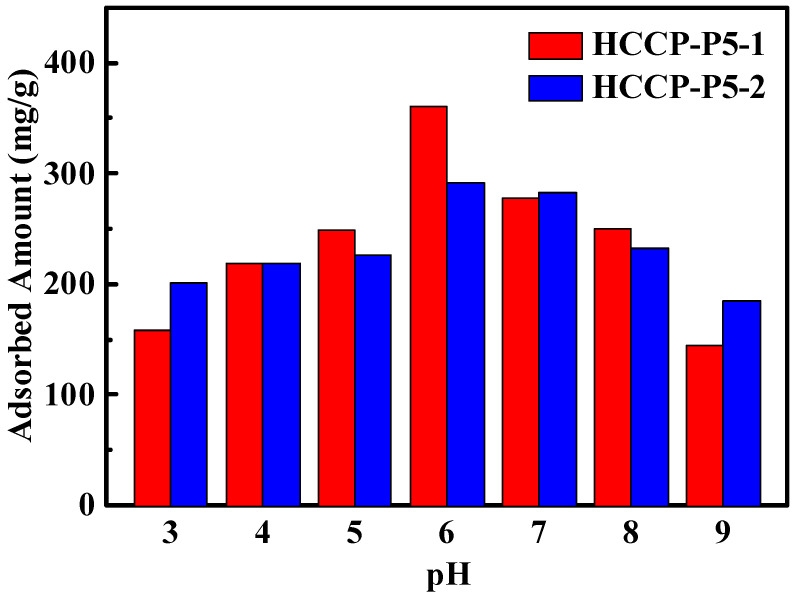
Effect of pH on uranium sorption on HCCP-P5-1 and HCCP-P5-2. (C_0_ = 9.25 ppm, V = 10 mL, T = 298.15 K, m = 5 mg).

The pH effect on the uranium adsorption capacity of both adsorbents HCCP-P5-1 and HCCP-P5-2 was investigated in the pH range from 3 to 9 which is presented in Figure 4. The adsorption ability of HCCP-P5-1 decreased from 360 mg/g to 150 mg/g at basic pH which revealed that deprotonating of the -OH groups of polymers occurred which decrease coordination capability with uranium. In contrast, HCCP-P5-2 is more stable, and the adsorption capacity change was recorded from 290 mg/g to 200 mg/g, which is indeed due to a smaller number of hydroxyl groups. Under acidic conditions, uranium is present in hydroxides and the prevalent cationic species are mainly UO_2_^2+^, UO_2_(OH)^+^, (UO_2_)_2_(OH)_2_^2+,^ and (UO_2_)_3_(OH)_5_ ^5+^. Under neutral conditions, uranium can be found in neutral species and starts to precipitate as UO_3_·2H_2_O and UO_2_(OH)_2_·H_2_O. Under basic conditions, uranium is mainly present in anionic species in the form of UO_2_(OH)^3−^ and UO_2_(OH)_4_^2−^. In acidic solutions, the hydrolysis process gradually occurs because of the abundance of OH^−^ ions in the solution and deprotonation takes place.

#### 2.2.2. Effect of Sorption Time and Kinetic Studies

**Figure 5:**
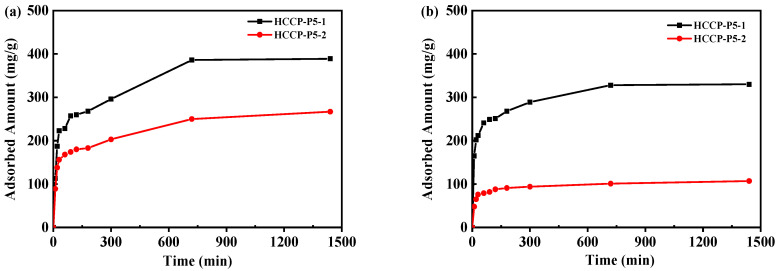
Effect of contact time on the uranium adsorption of HCCP-P5-1 and HCCP-P5-2 in (**a**) 9.25 ppm uranium-containing pure water and (**b**) 20 ppm uranium-containing simulated seawater. (V = 400 mL, T = 298.15 K, m = 5 mg).

The sorption kinetics of adsorbents were scrutinized by using uranium adsorption in pure water and seawater with diverse times at pH 6 as given in Figure 5. The pseudo-first, as well as second-order models, were used to study the controlled mechanism of the adsorption method. The equations are shown as under.

This is example 1 of the pseudo-first-order model equation:

(1) $$log\left(q_{e}-q_{t}\right)=logq_{e}-\frac{K_{1}t}{2.303}$$

This is example 2 of the pseudo-second-order model equation:

(2) $$\frac{t}{q_{t}}=\frac{1}{K_{2}q_{e}{}^{2}}+\frac{t}{q_{e}}$$

where *q_t_* denotes the sum of uranium (mg/g) sorption at time t and *q_e_* indicates the quantity of uranium (mg/g) sorption at equilibrium. *K*_1_ (min^−1^) and *K*_2_ (g/(mg·min) is the kinetic constants for the pseudo-first-order and pseudo-second-order models.

The experimental data of HCCP-P5-1 and HCCP-P5-2 were fitted by the two models. The respective kinetic constant values were presented in Table 2. (The fitted linear forms were displayed in Figures S5 and S6). In pure water, the R^2^ value of HCCP-P5-1 in pseudo-first-order and pseudo-second-order models are 0.810 and 0.995, respectively. Further, the values of R^2^ for HCCP-P5-2 in the two models are 0.821 and 0.997 whereas in the simulated seawater R^2^ values of HCCP-P5-1 in pseudo-first-order and pseudo-second-order models are 0.872 and 0.999, respectively. Moreover, the R^2^ value of HCCP-P5-2 in the two models is 0.930 and 0.999. These results suggested that the R^2^ value for the pseudo-second-order model is significantly high (R^2^ > 0.99). Hence, it is assumed that the uranium adsorption kinetics of HCCP-P5-1 and HCCP-P5-2 are pseudo-second-order processes and chemisorption can be the rate-controlling step.

#### 2.2.3. Effect of Initial Concentration and Isotherm Studies

**Figure 6:**
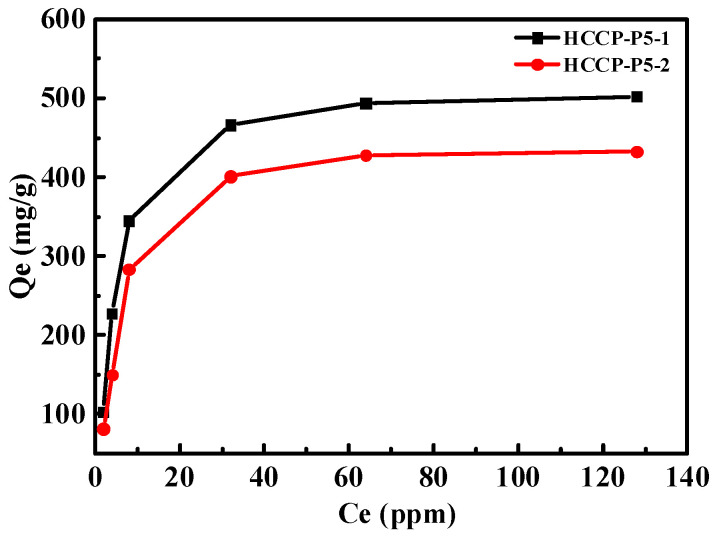
Initial concentration effect of uranium adsorption with HCCP-P5-1 and HCCP-P5-2. (V = 10 mL, T = 298.15 K, m = 5 mg).

The effect of the concentration of uranium and isotherm was investigated to gain more insight into the uranium adsorption capability of polymers, i.e., HCCP-P5-1 and HCCP-P5-2. As shown in Figure 6, initially sorption amounts of polymer HCCP-P5-1 were rising with the increasing concentration of C_0_ of uranium. After the uranium concentration reached 64 ppm, the sorption amounts of HCCP-P5-1 remained the same at 502 mg/g. Furthermore, the result for polymer HCCP-P5-2 was fully consistent with HCCP-P5-1 and recorded a maximum capacity of 432 mg/g. Further, Langmuir and Freundlich’s models are used to analyze the experimental data for describing the adsorption of solid to the liquid interface.

The Langmuir isotherm presumed that the sorption method is monolayer adsorption present at the particular homogenous site and its linear expression is shown in the given equation.

(3) $$q_{e}=\frac{q_{m}K_{L}C_{e}}{1+K_{L}C_{e}}$$

where the *q_e_* represents the amount of equilibrium sorption (mg/g). *C_e_* is the concentration of uranium (mg/L) at equilibrium while *q_m_* is the Langmuir monolayer saturated sorption amount (mg/L).

Furthermore, Freundlich’s model is an empirical calculation and exponential distribution of sorption’s site through the characteristic of heterogeneous surface and the linear formula which is given in the equation.

Where *K_F_* (mmol^1−1/n^L^1/n^g^−1^) is the constant for Freundlich’s adsorption capacity and n (unitless) is a constant for the adsorption intensity of the adsorbent. Langmuir and Freundlich’s model parameters of HCCP-P5-1 and HCCP-P5-2 are tabulated in Table 3 (fitted linear forms as seen in Figure S7).

The maximum theoretical sorption amount of the Langmuir model for HCCP-P5-1 and HCCP-P5-2 were calculated as 537.81 mg/g and 473.32 mg/g, respectively. The comparative study of the two models showed the R^2^ value of Langmuir’s model is higher (R^2^ > 0.99) which demonstrated that the adsorption route is monolayer whereas the adsorption sites on the surface of the materials are homogeneous.

#### 2.2.4. Mechanism of Uranium Sorption

**Figure 7:**
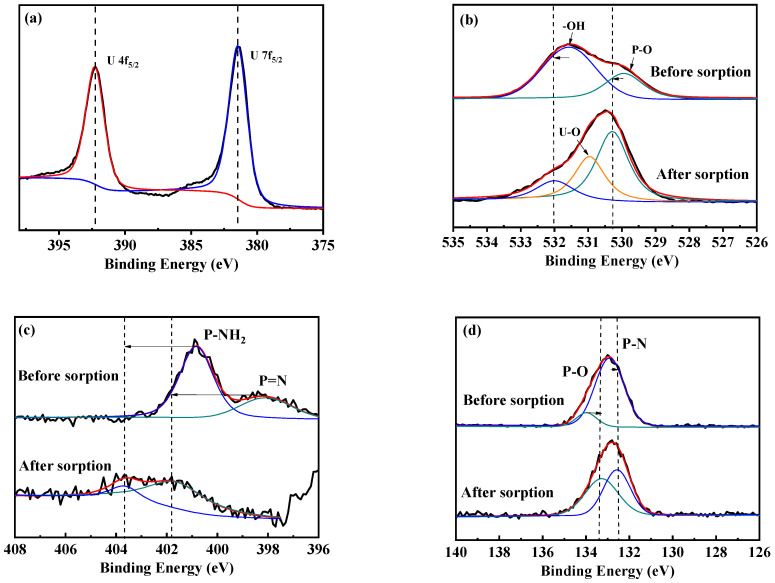
The XPS high-resolution spectra of HCCP-P5-1 before and after sorption of uranium: (**a**) U4f, (**b**) O1s, (**c**) N1s, and (**d**) P2p.

**Figure 8:**
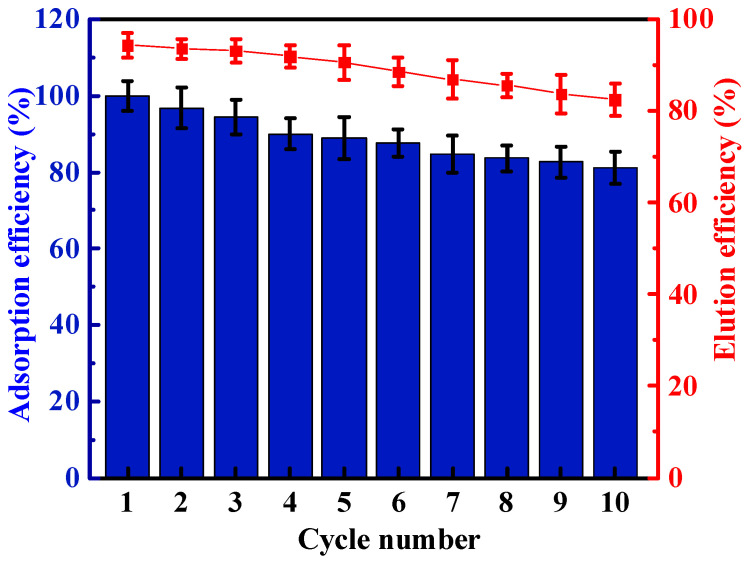
Effects of desorption on uranium adsorption behavior of HCCP-P5-2. (C_0_ = 9.25 ppm, V = 500 mL, T = 298.15 K, m = 5 mg).

FT-IR and XPS analysis were used to elucidate the variations of chemical composition and boding arrangements of HCCP-P5-1 and HCCP-P5-2 earlier and after the adsorption. For both polymers, a diagnostic new peak of the U–O bond was observed in the infra-red spectrum (Figure S8) and robust U 4f peaks were noticed in the XPS spectrum (Figures S8 and S9a) after sorption. This result validated that certain adsorption of uranium occurred on HCCP-P5-1 and HCCP-P5-2. The comparison of the electronic binding energy of elements showed no substantial shifts except O, N, and P. Furthermore, the high-resolution O 1s analysis shown in Figure 7b confirmed the existence of two species of oxygen in HCCP-P5-1 before sorption. The peaks exhibited at 530.0 eV and 531.6 eV could attribute to unreacted –OH and P–O bonds, respectively. After the sorption of uranium, electron binding energies of the two chemical states increased by 0.33 and 0.4 eV, respectively, and a new U–O peak seemed at 530.9 eV. Moreover, the high-resolution N 1s spectra (Figure 7c) showed two sorts of nitrogen species in HCCP-P5-1. The signal at 398.2 corresponds to P=N whereas the peak at 400.9 eV belongs to the P–NH_2_ which is formed via the isomerization of phosphazene. After the uranium sorption, the binding energy of electrons of both nitrogen species is enhanced by 2.6 eV and 1.8 eV, respectively. As shown in Figure 8d, the high-resolution P 2p spectra exhibited significant peaks at 132.9 eV and 133.9 eV, which can be attributed to the synthetic P–N and P–O bonds correspondingly. Further, after the uranium sorption, the electronic binding energies of species were decreased by 0.4 and 0.5 eV due to the inductive effect. Likewise, the FT-IR spectra of HCCP-P5-2 after sorption showed a consistent result as the electronic binding energies of O and N increased and P decreased noticeably (Figure S10). Therefore, both FT-IR and the XPS analysis certified that HCCP-P5-1 and HCCP-P5-2 have strong connections with uranyl ions via the N and O atoms.

#### 2.2.5. The Recyclability of the HCCP-P5-2

To illustrate the recyclability of both polymers, HCCP-P5-2 was taken as an example. An eluent solvent with ultrapure water, 30% aqueous hydrogen peroxide solution, and sodium bicarbonate powder was prepared to recycle HCCP-P5-2. After five runs, the adsorption capability of the HCCP-P5-2 decreased slightly to 88.9% in an aqueous solution (Figure 8). Even ten cycles later, HCCP-P5-2 remained at 81.0% adsorption efficiency and 82.5% elution efficiency, indicating that the HCCP-P5-2 showed high adsorption performance with a robust structural framework. The loss of some of the adsorbent during the experiment became the main reason for the decrease in adsorption capacity. These results implied that the HCCP-P5-2 could be used as an excellent adsorbent with a cost-effective strategy for the extraction and recovery of uranium.

#### 2.2.6. The Uranium Sorption Proficiencies of Other Reported POPs

The adsorption capacities of several representative materials in the uranium solution were compared and presented in Table 4. The results illustrated that the adsorption capability of HCCP-P5-1 in solution was superior as compared to reported COF or POPs adsorption materials.

## 3. Materials and Methods

### 3.1. Materials and Chemicals

P5 macrocycle was synthesized according to the method reported previously [36]. The required chemicals and solvents were acquired from authentic suppliers and utilized without further purification.

### 3.2. Synthesis of Polymer HCCP-P5-1 and HCCP-P5-2

P5 (2.0 mmol) and HCCP (2.0 mmol) were dissolved with 2 mL of dioxane solvent in a Schlenk bottle. Then, 1.68 mL trimethylamine was added, and the mixture was stirred for 24 h at 80 °C under the nitrogen condition. When the reaction has accomplished, the mixture was cooled down, filtered, and washed frequently with deionized water, ethanol, and acetone successively. Further, the product was dried in the vacuum oven overnight at 50 °C. Finally, HCCP-P5-1 was obtained with more than 55% yield. Moreover, a similar synthetic procedure has been adopted for the synthesis of the HCCP-P5-2 compound except that the ratio between P5 and HCCP was changed from 1:1 to 3:5 to decrease the number of free hydroxyl groups.

### 3.3. Materials Characterizations

The Bruker Avance II 400 instrument (Bruker BioSpin, Billerica, MA, USA) and CD_3_OD, DMSO-d_6_, and CDCl_3_ solvents were used to study ^1^H NMR spectroscopy while ^13^C NMR spectra were investigated by using a Bruker Av500 NMR spectrometer at 126 MHz. The FT-IR study was examined by a JASCO IR-4100 spectrometer (Jasco Int. Co. Ltd., Tokyo, Japan). HRMS (high-resolution mass) analyses were deliberate with a mass spectrometer (Agilent G6224A-TOF, Agilent Technologies UK Ltd., Stockport, UK). The solid-state ^13^C cross-polarization with magic angle spinning (CP/MAS) results were collected by a 500 MHz nuclear magnetic resonance spectrometer (Agilent DD2). X-ray diffraction (XRD) results were obtained by using diffractometers called Rigku D/max-2400 (40 kV, 200 mA) from 2° to 40° with a scanning rate of 2°/min. The FE-SEM analysis was examined by using an FEI Nova Nano SEM 450 scanning electron microscopy (Thermo Fisher Scientific, Inc., Waltham, MA, USA). The results of the adsorption and desorption of gases were obtained by an analyzer named Quantachrome Autosorb iQ (Quantachrome Instruments, Anton Paar, Graz, Austria). The TGA (thermogravimetric) analysis was achieved by the thermal analyzer (Mettler Toledo TGA/DSC 3+, Mettler Toledo, Zurich, Switzerland) under the N_2_ condition. Samples were heated from 25–600 °C with a 10 °C/min heating rate. XPS spectroscopy (X-ray photoelectron, Thermo Scientific ESCALAB 250Xi) was utilized to evaluate the elemental species on the surface of the materials.

### 3.4. Sorption Experiments

The stock solutions of uranium with different concentrations in pure water and simulated seawater were prepared. The pH of the solutions was adjusted by 3M HNO_3_ or 1M NaOH solution. The concentrations of uranium during experiments were detected via ICP-OES (Inductively coupled plasma optical emission) spectroscopy while inductively coupled plasma mass spectrometry (ICP-MS) was used for extra-low concentrations. The adsorption experiments were performed under ambient conditions. In addition, the uranium solution without sorbent was scrutinized for respective sorption experiments as a negative control. The same experiment was repeated three times and the final results were obtained by taking the average value.

#### 3.4.1. Uranium Sorption Isotherms

To acquire the uranium adsorption isotherms for two adsorbents, 5 mg of HCCP-P5-1 or HCCP-P5-2 were mixed into aqueous solutions of uranium (10 mL) with various concentrations. Adsorbents were fully suspended by short sonication and the mixtures were vigorously stirred overnight. The solutions were filtered via a 0.45 µm membrane filter. The supernatant was evaluated by using ICP analysis to find the concentration of uranium remains. The adsorbed quantity at equilibrium (QE, mg g^−1^) was attained in the given equation.

(4) $$q_{e}=\frac{\left(C_{0}-C_{e}\right)V}{m}$$

Wherever *V* is the volume of the treated solution (mL), *m* is the amount of used adsorbent (g), *C*_0_ and *C_e_* are the initial concentration and equilibrium concentration of uranium correspondingly.

#### 3.4.2. Uranium Adsorption Kinetics from U-Spiked Pure Water

Uranium aqueous solution (400 mL, 9.25 ppm) and adsorbent (5 mg) were added to an Erlenmeyer flask. The mixture was stirred for 3 h at 25 °C. At appropriate time intervals, aliquots (5 mL) were taken from the mixture, and the adsorbents were filtered by a syringe with a 0.45 μm membrane filter. The uranium concentrations in the resulting solutions were analyzed with ICP-OES.

#### 3.4.3. Uranium Adsorption Kinetics from U-Spiked Simulated Seawater

An amount of 200 mL of the simulated seawater spiked with uranium (20 ppm) and 5 mg adsorbents were mixed in the Erlenmeyer flask. The reaction mixture was strongly stirred at 25 °C. After the reaction time, 5 mL of the aliquots were taken from the mixture then the adsorbents were filtered by a 0.45 μm membrane filter syringe. The concentrations of uranium in the resulting solutions were examined by using ICP-OES.

#### 3.4.4. The Recyclability of the Sample

A 5 mg sample was immersed and shaken in 500 mL of 16 ppm U-spiked water (pH = 6) for 48 h. Further, the U-uptake sample was immersed and shaken in a 100 mL eluent for 30 min. The eluent solution was prepared with 1000 mL ultrapure water, 11.4 mL 30% aqueous hydrogen peroxide solution, and 106 g sodium bicarbonate powder. The U-adsorption and the U-desorption amount can be calculated on sed the U-concentration change in the U-spiked water and eluent solution, respectively.

## 4. Conclusions

In this research, two new pillar[5]arene- and phosphazene-linked POPs, HCCP-P5-1, and HCCP-P5-2 were successfully constructed by a macrocycle-to-framework strategy using P5 macrocycle as the functional monomer and hexachlorophosphates (HCCP) as the linker. The isothermal adsorption curves and kinetic studies showed that the adsorption of POPs on uranium was consistent with the Langmuir model and the pseudo-second-order kinetic model. Both materials displayed a high-efficient uranium adsorption capacity of 537.81 mg/g of HCCP-P5-1 and 473.32 mg/g of HCCP-P5-2 which were much better performances than the previously reported POPs adsorption materials to date, especially those non-macrocycle-incorporated counterparts. In addition, the stability and adsorption capacity of both polymers can be regulated by the monomer ratio between P5 and HCCP. HCCP-P5-1 with more P5 has higher uranium adsorption capacity, but HCCP-P5-2 with a higher HCCP ratio has stronger stability under the conditions of strong acid and alkali. Even ten cycles later, HCCP-P5-2 remained at 81.0% adsorption efficiency and 82.5% elution efficiency, which is more suitable for a complex realistic environment. This work is expected to promote the application of pillararene-based porous polymers in the field of uranium adsorption and the macrocycle-to-framework strategy could provide new insight into the design and construction of highly efficient uranium adsorbents.

## Figures and Tables

**Table 1:** Textural and sorption performance.

POPs	S_BET_ (m^2^/g)	V_Total_ (cc/g) ^a^	Pore Size (nm)
HCCP-P5-1	17.1	0.043	4.728
HCCP-P5-2	23.3	0.058	4.728

^a^ Total pore volume calculated from nitrogen isotherm at P/P_0_ = 0.99.

**Table 2:** The data were evaluated from pseudo-first-order and pseudo-second-order.

Solution	Polymers	Pseudo-First-Order Model			Pseudo-Second-Order Model		
		Q_e_ (mg/g)	K_1_	R^2^	Q_e_ (mg/g)	K_2_	R^2^
Pure water	HCCP-P5-1	310.23	0.037	0.810	401.60	4.95 × 10^−5^	0.995
	HCCP-P5-2	207.64	0.047	0.821	271.73	7.50 × 10^−5^	0.997
Simulated seawater	HCCP-P5-1	279.37	0.064	0.872	335.57	1.09 × 10^−4^	0.999
	HCCP-P5-2	92.27	0.062	0.930	107.64	3.76 × 10^−4^	0.999

**Table 3:** Value of parameters for langmuir and freundlich.

Polymers	Langmuir Isotherm			Freundlich Isotherm		
	Q_m_ (mg/g)	K_L_	R^2^	K_F_	N	R^2^
HCCP-P5-1	537.81	0.0986	0.976	170.28	0.245	0.793
HCCP-P5-2	473.32	0.0696	0.973	128.81	0.274	0.799

**Table 4:** Comparisons of uranium adsorption capacities of different porous organic adsorbents.

No	Adsorption	q_m_ (mg/g)	pH	Ref.
1	CCOF-SCU1	50	1.0	[43]
2	COF TCD	158	4.5	[44]
3	MPCOF	214	4.5	[10]
4	COF-TpAb-AO	408	6.0	[13]
5	COF-HBI	211	4.5	[45]
6	COF-IHEP1	112	5.0	[46]
7	PAF-1-CH2AO	300	——	[47]
8	TFPT-BTAN-AO	427	4.0	[48]
9	COF-SO3H	360	5.0	[49]
10	COF-HAP	510	3.0	[50]
11	HCCP-P5-1	537	6.0	This work
12	HCCP-P5-2	473	6.0	This work

## Data Availability

All data related to this study are presented in this publication.

## Supplementary Materials

The following supporting information can be downloaded at: https://www.mdpi.com/article/10.3390/molecules28031029/s1, Scheme S1: Synthetic pathway for the preparation of monomers pillar[5]arene. Figure S1: TGA curves of HCCP-P5-1 and HCCP-P5-2. Figure S2: SEM micrographs of (a) HCCP-P5-1 and (b) HCCP-P5-2. Figure S3: XRD patterns of HCCP-P5-1 and HCCP-P5-2. Figure S4. Pore size distribution of HCCP-P5-1 and HCCP-P5-2 from the N_2_ adsorption isotherm. Figure S5. The fitted linear forms of (a) pseudo-first-order kinetic model and (b) pseudo-second-order kinetic model of HCCP-P5-1 and HCCP-P5-2 in pure water. Figure S6. The fitted linear forms of (a) pseudo-first-order kinetic model and (b) pseudo-second-order kinetic model of HCCP-P5-1 and HCCP-P5-2 in the seawater. Figure S7. The Langmuir isotherm fitted linear forms and the Freundlich isotherm fitted linear forms of HCCP-P5-1 and HCCP-P5-2. Figure S8. The XPS spectra of (a) HCCP-P5-1 and (b) HCCP-P5-2. Figure S9. The XPS high-resolution spectra of HCCP-P5-2 before and after sorption of uranium: (a) U4f, (b) O1s, (c) N1s, and (d) P2p. Figure S10 The FT-IR of HCCP-P5-1 and HCCP-P5-2 before and after adsorption of UO^2+^.

## Sample Availability

Samples of the compounds are not available from the authors.
