# Supplementary material for: Synthesis of Pillar[5]arene- and Phosphazene-Linked Porous Organic Polymers for Highly Efficient Adsorption of Uranium
Source: Molecules. 2023 Jan 19;28(3):1029. doi: 10.3390/molecules28031029 (PMC9920965; doi:10.3390/molecules28031029)
Supplement: Supplementary file 1 [file molecules-28-01029-s001.zip › molecules-2150676-supplementary.pdf]

molecules

MDPI

Supplementary Materials

Synthesis of pillar [5]arene- and phosphazene-linked porous organic polymers for highly efficient adsorption of uranium

Xiaoxiao Zhao [1], Ziyi Liu [1], Shuguang Zhang [1], Mehdi Hassan [2], Chunxin Ma [3,4,*], Zhenzhong Liu [4] and Weitao Gong [1,*]

1 School of Chemical Engineering, Dalian University of Technology, Dalian 116024, China; zxx1922@163.com (X.Z.); liuzy2020@mail.dlut.edu.cn (Z.L.); zxx1001@mail.dlut.edu.cn (S.Z.)
2 Department of Chemistry, University of Baltistan, Skardu 16100, Pakistan; mehdi.hassan@uobs.edu.pk
3 State Key Laboratory of Marine Resource Utilization in South China Sea, Hainan University, Haikou 570228, China
4 Research Institute of Zhejiang University-Taizhou, Taizhou 318000, China; zzliu@zju.edu.cn (Z.L.)
* Correspondence: machunxin@hainanu.edu.cn (C.M.); wtgong@dlut.edu.cn (W.G.); Tel.: +159-0426-8498 (W.G.)

1. Synthesis of dimethoxypillar[5]arene and pillar[5]arene[1]

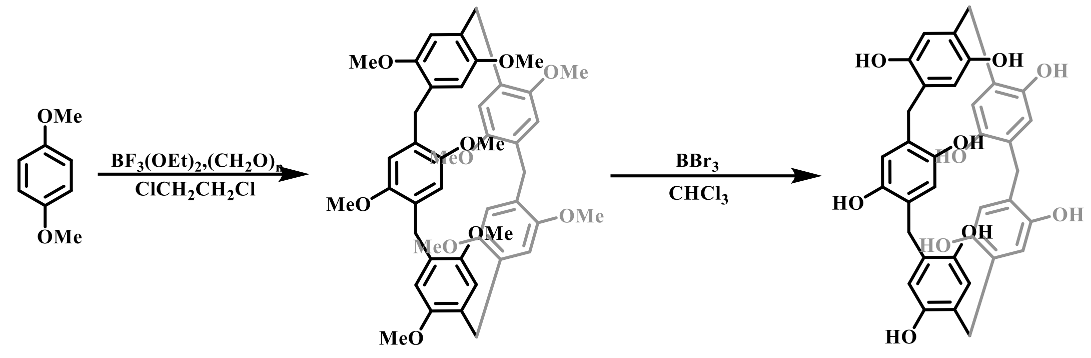

Scheme S1. Synthetic pathway for the preparation of monomers pillar [5] arene.

To a solution of 1,4-dimethoxybenzene (1.38 g, 10 mmol) in 1,2-dichloroethane (20 mL), paraformaldehyde (0.31 g, 10 mmol) was added under nitrogen atmosphere. The reaction flask was capped, and nitrogen bubbled through the solution for 30 minutes. Then, boron trifluoride diethyl etherate [$BF_3{\cdot}O(C_2H_5)_2$, 1.25 mL, 10 mmol] was added to the solution and the mixture was stirred at room temperature for 3 h. The solution was poured into methanol and the resulting precipitate was collected by filtration. The obtained solid was re-crystallized from acetonitrile to yield 0.33 g of DMpillar[5]arene as a while solid. Yield: 30.0%.

To a solution of DM-pillar [5] arene (0.34 g, 0.40 mmol) in chloroform (20 mL), boron tribromide (2.40 g, 9.60 mmol) was added. The mixture was stirred at room temperature for 96 h. Then, the solid formed during the reaction was collected by filtration and washed with water. Column chromatography (silica gel, 50% CH_2Cl_2 / 50% acetone) afforded a white solid (pillar [5] arene, 0.072 g, 0.12 mmol). Yield: 62%.

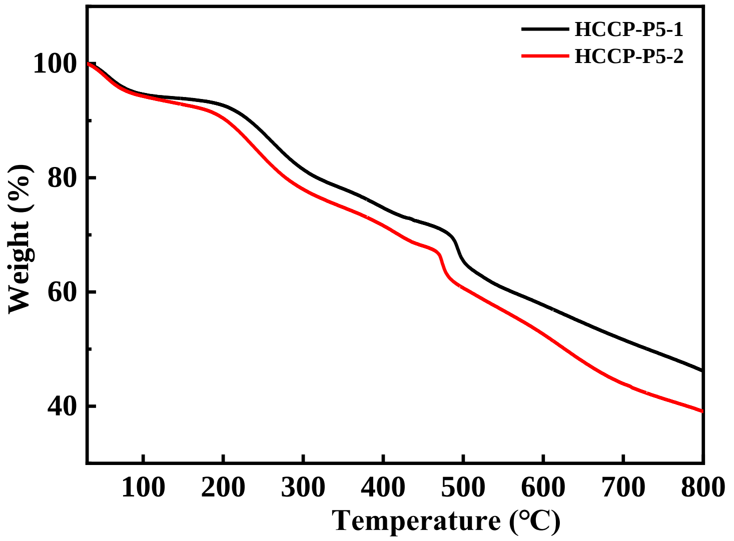

Figure S1. TGA curves of HCCP-P5-1 and HCCP-P5-2.

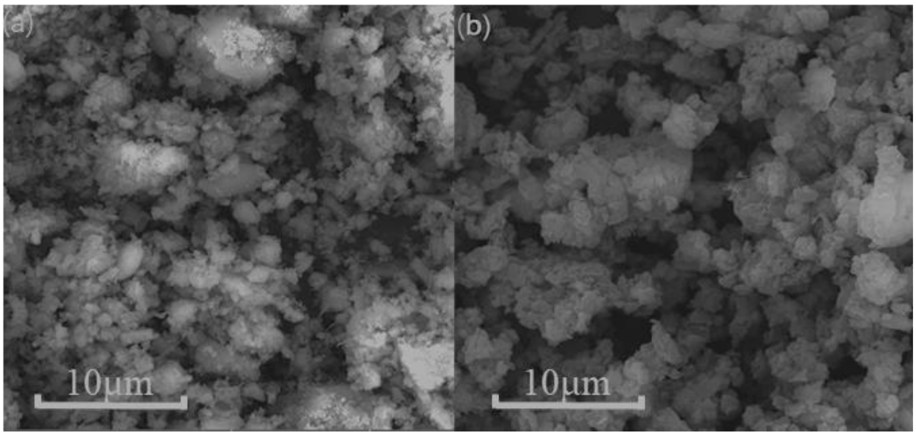

Figure S2. SEM micrographs of **(a) HCCP-P5-1** and **(b) HCCP-P5-2.**

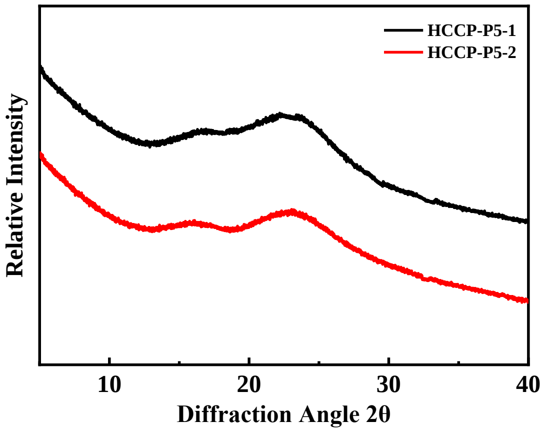

Figure S3. XRD patterns of **HCCP-P5-1 and HCCP-P5-2.**

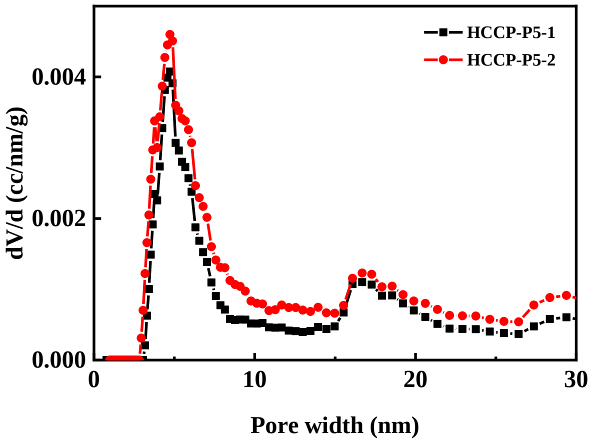

Figure S4. Pore size distribution of **HCCP-P5-1** and **HCCP-P5-2** from the N_2 adsorption isotherm.

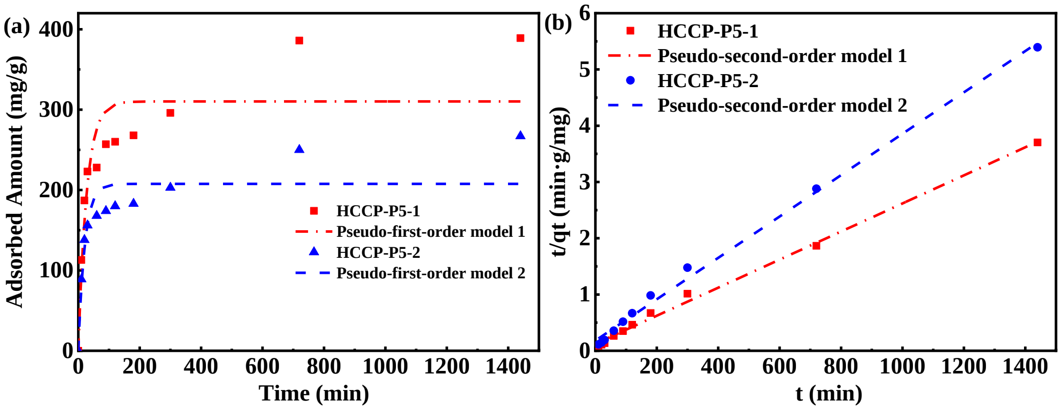

Figure S5. The fitted linear forms of **(a)**pseudo-first-order kinetic model and **(b)** pseudo-second-order kinetic model of **HCCP-P5-1** and **HCCP-P5-2** in the pure water.

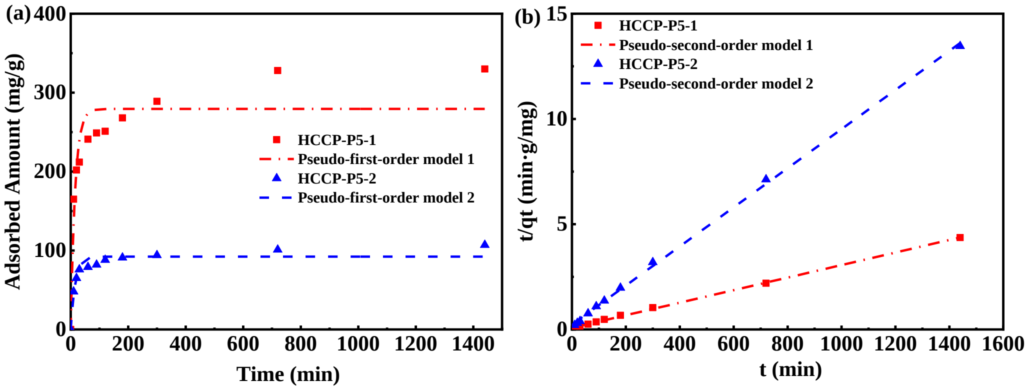

Figure S6. The fitted linear forms of (**a**) pseudo-first-order kinetic model and (**b**) pseudo-second-order kinetic model of **HCCP-P5-1** and **HCCP-P5-2** in the sea water.

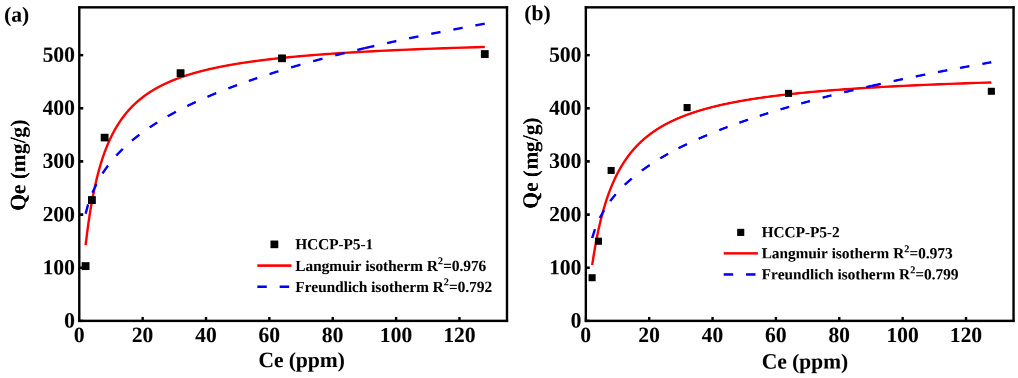

Figure S7. The Langmuir isotherm fitted linear forms and the Freundlich isotherm fitted linear forms of **HCCP-P5-1** and **HCCP-P5-2.**

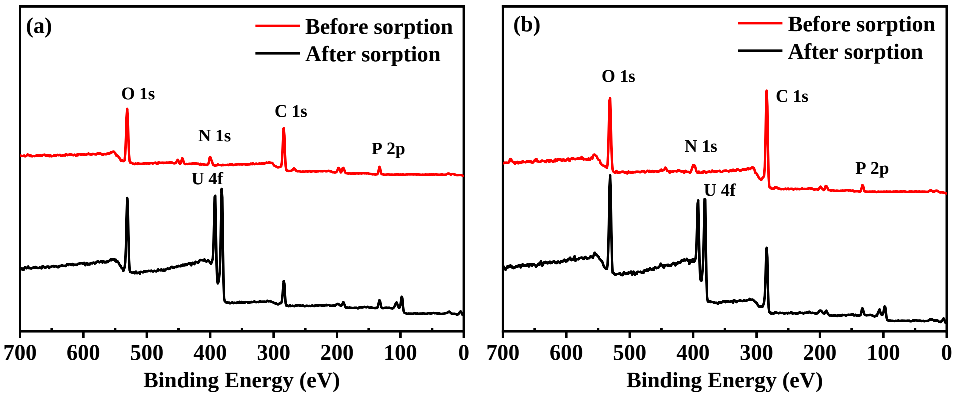

Figure S8. The XPS spectra of (**a**) **HCCP-P5-1** and (**b**) **HCCP-P5-2.**

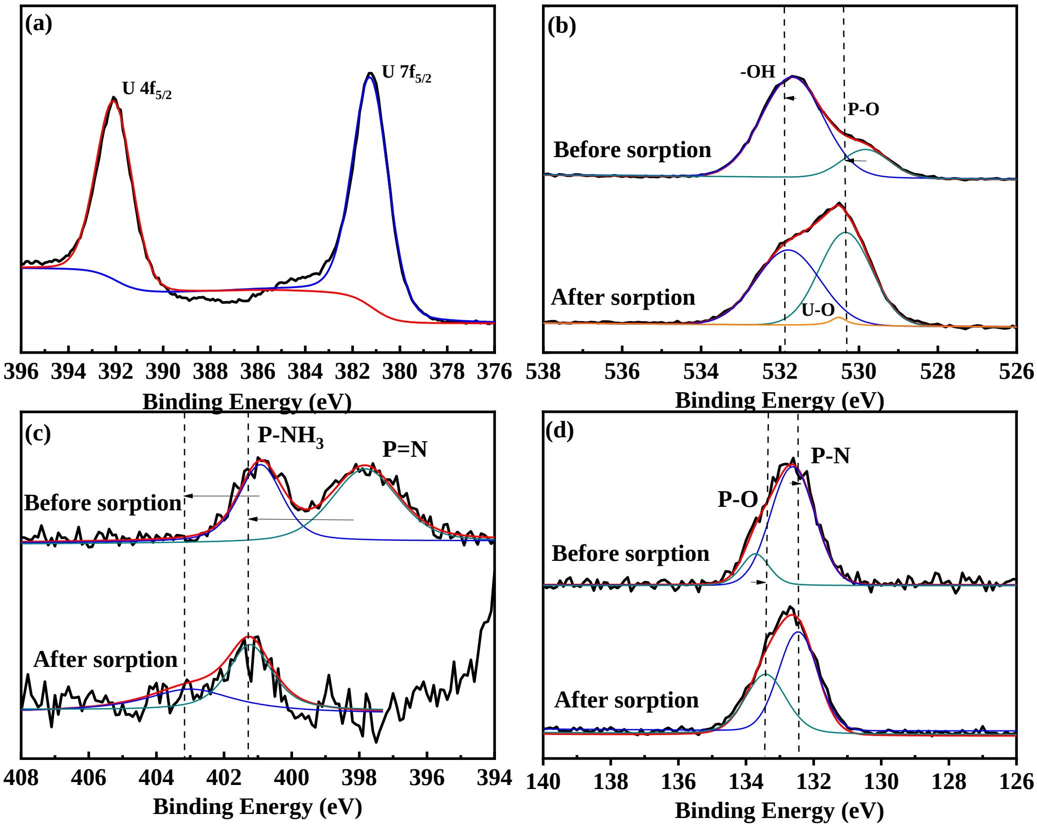

Figure S9. The XPS high-resolution spectra of **HCCP-P5-2** before and after sorption of uranium: (**a**) U4f, (**b**) O1s, (**c**) N1s and (**d**) P2p.

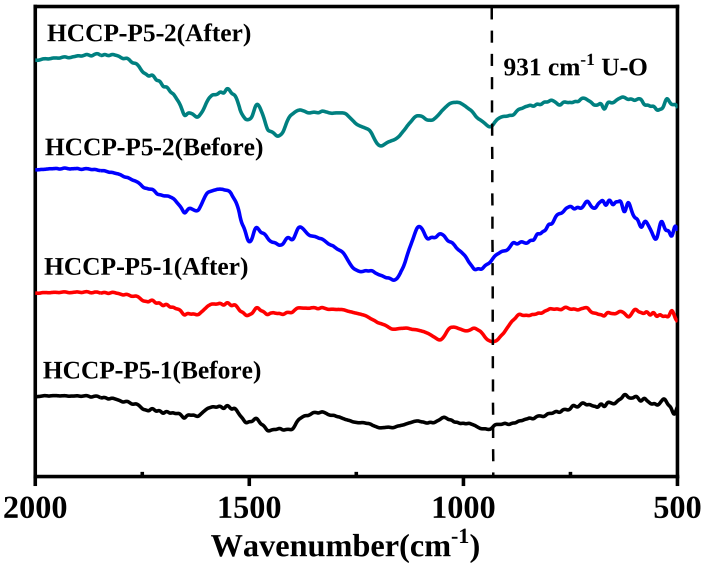

Figure S10 The FT-IR of **HCCP-P5-1** and **HCCP-P5-2** before and after adsorption of uranium.

T. Ogoshi., S. Kanai., S. Fujinami., T.-a. Yamagishi., Y. Nakamoto., 2008. para-Bridged Symmetrical Pillar[5]arenes: Their Lewis Acid Catalyzed Synthesis and Host–Guest Property, J. Am. Chem. Soc. 13, 5022-5023.
